# Protein Intake and Distribution in Relation to Physical Functioning and Quality of Life in Community-Dwelling Elderly People: Acknowledging the Role of Physical Activity

**DOI:** 10.3390/nu10040506

**Published:** 2018-04-19

**Authors:** Dominique S. M. ten Haaf, Ellen J. I. van Dongen, Malou A. H. Nuijten, Thijs M. H. Eijsvogels, Lisette C. P. G. M. de Groot, Maria T. E. Hopman

**Affiliations:** 1 Department of Physiology (392), Radboud University Medical Center, P.O. Box 9101, 6500 HB Nijmegen, The Netherlands; dominique.tenhaaf@radboudumc.nl (D.S.M.t.H.); Malou.Nuijten@radboudumc.nl (M.A.H.N.); Thijs.Eijsvogels@radboudumc.nl (T.M.H.E.); 2 Wageningen Food & Biobased Research, Food, Health & Consumer Research, P.O. Box 17, 6700 AA Wageningen, The Netherlands; Ellen.vanDongen@wur.nl; 3 Division of Human Nutrition, Wageningen University, P.O. Box 17, 6700 AA Wageningen, The Netherlands; Lisette.deGroot@wur.nl

**Keywords:** protein intake, protein intake distribution, physical activity, physical functioning, community-dwelling, elderly

## Abstract

Increasing total protein intake and a spread protein intake distribution are potential strategies to attenuate sarcopenia related loss of physical function and quality of life. The aim of this cross-sectional study was to investigate whether protein intake and protein intake distribution are associated with muscle strength, physical function and quality of life in community-dwelling elderly people with a wide range of physical activity. Dietary and physical activity data were obtained from two studies (N = 140, age 81 ± 6, 64% male), with the following outcome measures: physical functioning (Short Physical Performance Battery (SPPB), comprising balance, gait speed and chair rise tests), handgrip strength and quality of life (EQ-5D-5L). Protein intake distribution was calculated for each participant as a coefficient of variance (CV = SD of grams of protein intake per main meal divided by the average total amount of proteins (grams) of the main meals). Based on the CV, participants were divided into tertiles and classified as spread, intermediate or pulse. The average total protein intake was 1.08 ± 0.29 g/kg/day. Total protein intake was not associated with outcome measures using multivariate regression analyses. Individuals with a spread protein diet during the main meals (CV < 0.43) had higher gait speed compared to those with an intermediate diet (CV 0.43–0.62) (*β* = −0.42, *p* = 0.035), whereas a spread and pulse protein diet were not associated with SPPB total score, chair rise, grip strength and Quality-Adjusted Life Year (QALY). The interaction of higher physical activity and higher total protein intake was significantly associated with higher quality of life (*β* = 0.71, *p* = 0.049). While this interaction was not associated with SPPB or grip strength, the association with quality of life emphasizes the need for a higher total protein intake together with an active lifestyle in the elderly.

## 1. Introduction

Sarcopenia is defined as the age-related loss of muscle mass and muscle strength, resulting in impaired physical function [1] and loss of independence for daily life activities [2], which is associated with a decreased quality of life and an increased health care expenditure [3,4]. On average, 5–13% of the elderly aged 60–70 years are affected by sarcopenia with prevalence increasing to 11–50% in elderly people over the age of 80 years [5]. Therefore, it is important to identify strategies to counteract sarcopenia.

Sufficient protein intake is essential for muscle protein synthesis and the consequent preservation or improvement of muscle mass and strength [6]. Based on the age-related decline in protein utilization for muscle protein synthesis [7,8,9], Bauer et al. proposed a protein intake for the elderly of 1.0–1.2 g protein per kilogram body weight per day (g/kg/day) [6], a dose which is well above the current recommendations of 0.8 g/kg/day for all adults [10,11].

Besides the amount of protein intake, the protein intake distribution might be associated with muscle mass and strength. Some studies support a pulse-feeding pattern in which a high protein meal might saturate the splanchnic sequestration leading to a higher availability of amino acids for muscle protein synthesis [12,13]. In contrast, several other studies reported an optimized muscle protein synthesis using a more continuous availability of amino acids in a spread-feeding pattern [14,15,16,17,18]. As yet, the current literature is not conclusive about the most efficient protein intake distribution for optimal muscle protein synthesis.

Another strategy to influence muscle strength as well as physical function in the elderly is to enhance physical activity [19,20]. In addition to the independent effect of physical activity on muscle strength, previous literature reported an exercise-induced increase in anabolic sensitivity to dietary protein for up to 24 h after exercise [21]. It seems therefore of utmost importance to take physical activity of the individual into account when studying the effect of protein intake and distribution on muscle characteristics. Unfortunately, most studies do not include physical activity in the equation [12,13,15].

Therefore, the aim of this study is to investigate whether protein intake and protein intake distribution are associated with muscle strength, physical function and quality of life in community-dwelling elderly people additionally accounting for the role of physical activity. We hypothesize that higher protein intake and a spread protein distribution are associated with improved strength and physical function, while adding physical activity to the equation will enlarge these effects.

## 2. Methods

### 2.1. Study Population

This study included data collected from 140 adults aged 65 years or older. Participants were from two studies, thus creating a wide range of physical activity. The first sample included participants of the Nijmegen Four Days Marches. These elderly people were approached via mail and a total of 82 participants were included. To include a sample of less active elderly people, baseline data of participants from the ProMuscle in Practice study (*n* = 58) were included. Participants were mainly recruited through local media outings, flyers and home care providers. This trial was registered in the Netherlands Trial Register (NTR6038). Both studies were approved by a local Medical Ethical Committee (CMO registration number: 2007/148 and 16/12, respectively), conducted in accordance with the Declaration of Helsinki and all participants gave written informed consent prior to participation.

### 2.2. Study Design

In the present cross-sectional study, we measured protein intake, physical activity, muscle strength, physical functioning and quality of life in 140 participants from two distinct studies. Eighty-two participants were recruited within the Four Days Marches study. Measurements were performed one or two days prior to the Four Days Marches, while the participants’ habitual dietary intake was assessed one month later to make sure their intake was representative of a regular period of the year. The remaining 58 participants were recruited within the ProMuscle in Practice study, whereas all measurements were performed before participants started the intervention.

### 2.3. Measurements

#### 2.3.1. Protein Intake

In the Four Days Marches study, daily dietary intake was assessed using two 24-h recalls, a validated method for assessing the amount and distribution of protein intake [22]. The two recall days were randomized over the week with the restriction that no participant was assigned to two identical week days or to two weekend days. The 24-h recalls were performed face-to-face or by phone by trained dieticians. Portion sizes were documented in household measures, whereby frequently used household measures were subsequently quantified with standard portion sizes. In the ProMuscle in Practice study, dietary intake was assessed using three-day food records, which is another validated method to measure protein intake in the elderly [23]. Each participant was randomly assigned to two weekdays and one weekend day. Research dietitians gave oral and written instructions about completing the food record. After completion of the food record, a trained research dietitian visited the participants at home to check the food record. During this home visit, the dietitian also weighed and measured a standardized selection of food items and household measures that were linked to protein intake. De Keyzer et al. [24] reported a fair strength of agreement between the 24-h recalls and the food records for protein intake and concluded that group level intakes of protein did not differ [24]. Data were coded by trained dietitians and energy and macronutrient intake was calculated using Compl-eat, based on the Dutch food composition table (NEVO, 2013) [25]. The mean of the recorded days was calculated for total daily intake and intake per main meal (breakfast, lunch, dinner).

#### 2.3.2. Evaluation of Underreporting Energy Intake

Underreporting of energy intake (EI) of the participants was evaluated with Goldberg’s method, which is based on the ratio of energy intake and basal metabolic rate (BMR) [26]. BMR was calculated using Schofield’s equations, which is based on age, body weight and height [27]. To set a Goldberg cut–off value to identify underreporting or overreporting, we assumed a within–subject variation in energy intake of 23%, a within–subject variation in estimated BMR of 8.5%, a physical activity level of 1.55 and a between–subject variation in physical activity level of 15% [26,28,29]. For participants ≥70 years, a Goldberg score of <0.89 was defined as underreporting and >2.66 as overreporting [29]. Analysis were performed with and without inclusion of participants that were under- or overreporting and when results were different, both were reported.

#### 2.3.3. Physical Activity

In the Four Days Marches study, physical activity was assessed by the Short Questionnaire to Assess Health enhancing physical activity (SQUASH), which is considered a valid and reliable method in the elderly [30]. This self-administered questionnaire estimates habitual level of physical activity during a normal week over the past month. In the ProMuscle in Practice study, physical activity was assessed using the Longitudinal Aging Study Amsterdam physical activity questionnaire (LAPAQ), which is another valid method to assess physical activity in the elderly [31]. It measures physical activities performed in the past two weeks and was completed together with a researcher.

Both questionnaires include walking, cycling, gardening, light and heavy household activities and sports activities. Information was collected about type, duration and frequency of these activities. The intensity for each activity was determined based on activity intensity classification according to Ainsworth’s Compendium of Physical Activities [32]. Total physical activity and activity-specific activity could be calculated in MET-hours per day (METhr/day) by multiplying the exercise time in hours with the accompanying MET score of the activity intensity [32].

#### 2.3.4. Muscle Strength

In both studies, muscle strength was measured by handgrip strength of the dominant hand. This was measured with a hydraulic, analogue hand dynamometer (Jamar, Jackson, MI, USA). For every participant, the dynamometer was adjusted to their hand size. The participants were seated in a chair with the elbow flexed in a 90-degree angle position. Arm support by the chair was not allowed. Three measurements were performed with approximately 30 s rest between measurements. The maximum strength effort in kilograms was used for analysis.

#### 2.3.5. Physical Functioning

In both studies, physical function was assessed by the Short Physical Performance Battery (SPPB), which is considered a reliable and valid method in the elderly [33]. The SPPB consists of three components: balance, gait speed and chair rise ability. In the balance test, participants were asked to stand still for 10 s in three positions: feet side by side, feet in semi-tandem position, feet in tandem position. Gait speed is determined by the time necessary to complete a walk of 4 m at normal gait speed. The chair rise ability score was determined by the time necessary to rise out of a chair and sit down five times in a row, without the aid of the arms. For each component, a score of 0–4 points could be earned. A SPPB total score (0–12 points) was calculated by summing up the scores of the three tests, in which a higher score reflects a better physical function.

#### 2.3.6. Quality of Life

Quality of life was measured in both studies using the EQ-5D-5L questionnaire. This five-item questionnaire includes the domains mobility, self-care, usual activities, pain and discomfort and anxiety/depression. Each question has five levels of functioning, ranging from no problems (1) to very severe problems (5). This questionnaire was used to calculate Quality-Adjusted Life Years (QALYs) [34]. Additionally, participants scored their current perceived health on a scale from 0 (worst imaginable health) to 100 (best imaginable health).

#### 2.3.7. Background Characteristics

Height and weight of each participant were measured and used to calculate BMI. Furthermore, additional questions about smoking, level of education and use of (vitamin D) supplements were included in the questionnaire.

#### 2.3.8. Statistical Analysis

The statistical analyses were performed using SPSS 22 software (IBM SPSS Statistics for Windows, Version 22 IBM Corp., Armonk, NY, USA). All continuous variables were visually inspected and tested for normality with the Shapiro-Wilk test. Participant characteristics were displayed as means ± SDs or median (IQR) for parametric and non-parametric continuous variables respectively and as counts with percentages for categorical variables. First, participants were stratified into two groups based on protein intake with a cut-off 1.0 g/kg/day and differences between these groups were tested with an independent samples *t*-test for parametric variables, Kruskal-Wallis test for non-parametric variables, or Chi-square test for categorical data. Second, protein intake distribution was calculated for each participant as a coefficient of variance (CV = SD of grams of protein intake per main meal divided by the average total amount of proteins (grams) of the main meals). Based on the CV, participants were divided into tertiles. A low CV represents less difference in protein intake between the meals and therefore a more spread distribution, whereas a high CV represents a pulse-feeding distribution of protein intake. Differences between tertiles were tested using an ANOVA for parametric variables, a Kruskal-Wallis test for non-parametric variables and a Chi-square test for categorical data. Furthermore, the associations between protein intake, protein distribution and physical activity and physical function, muscle strength and quality of life were analyzed in a multivariate linear regression (forced entry method, including confounders age, sex, BMI and protein source). Statistical significance was assumed at *p* < 0.05 (two–sided).

## 3. Results

### 3.1. Descriptive Characteristics

A total of 140 participants (90 males and 50 females) with a median age of 83 years (interquartile range (IQR): 77–84) were included in the analysis (Table 1). Body mass index (BMI) was 25.9 ± 2.7 kg/m^2^ in males and 26.4 ± 4.7 kg/m^2^ in females. Habitual energy intake was 2040 ± 370 kcal for males and 1754 ± 396 kcal for females. Based on the Goldberg-cutoff there were no participants overreporting and five participants (3.6%) underreporting their energy intake. The average total protein intake was 79 ± 19 g/day—or 1.08 ± 0.29 g/kg/day when adjusted for bodyweight—and 62 ± 9% was animal source protein. Thirty participants (21%) used vitamin D containing supplements. Total physical activity was estimated at 8.4 METhr/day (IQR: 5.1–13.7), with most of the activities performed during leisure time, followed by household activities and sport activities (Table 1).

### 3.2. Total Protein Intake

A total of 80 participants consumed more protein in total than 1.0 g/kg/day, whereas 60 participants consumed less than 1.0 g/kg/day (Table 1). Participants with a higher total protein intake (HPI, ≥1.0 g/kg/day) did not significantly differ from participants with a lower total protein intake (LPI, <1.0 g/kg/day) with respect to age, sex, smoking behavior, level of education, vitamin D supplement use, physical activity, grip strength, SPPB scores or quality of life. In the regression analysis, total protein intake was not related to SPPB total score, gait speed, chair rise ability, handgrip strength or QALY (Table 2).

### 3.3. Protein Intake Distribution

**Figure 1:**
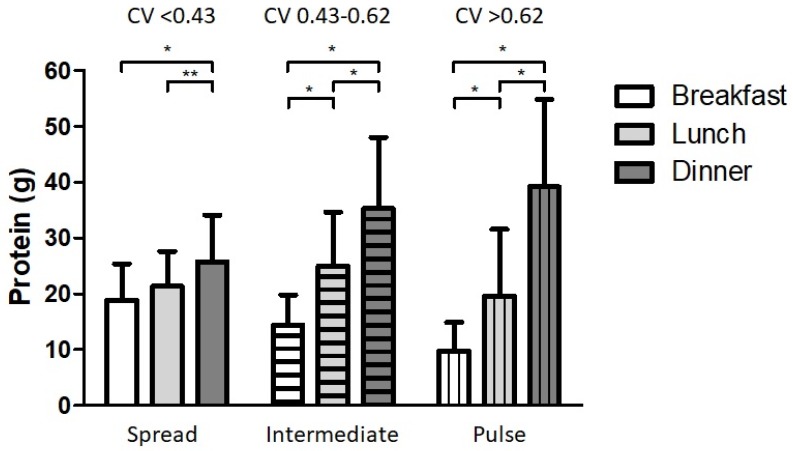
Protein intake during main meals of the participants in tertiles based on CV (coefficient of variance). Participants in the spread group (*n* = 46, CV < 0.43) had a significantly higher protein intake at dinner compared to the protein intake at breakfast and lunch. Participants in the intermediate group (*n* = 48, CV 0.43–0.62) and participants in the pulse group (*n* = 46, CV > 0.62) had significant different intakes at all main meals. * *p* < 0.001, ** *p* = 0.011. Data are presented as means ± SDs.

**Figure 2:**
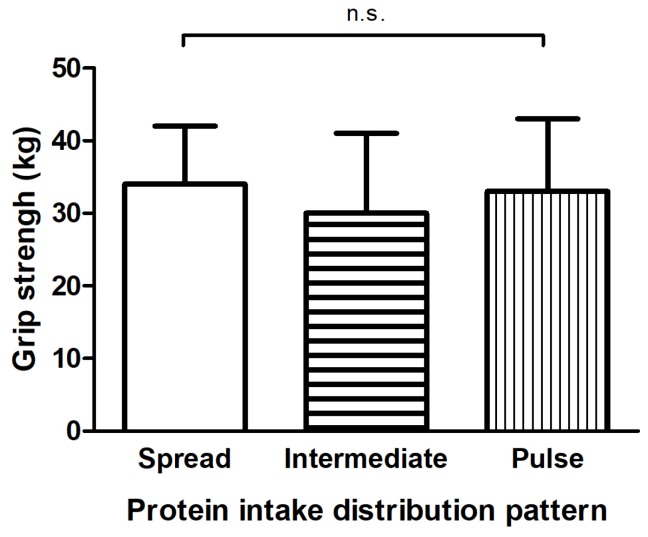
Hand grip strength of 3 groups based on distribution pattern of protein intake during the main meals determined with CV (coefficient of variance). Participants in the spread group (*n* = 46, CV < 0.43), intermediate group (*n* = 48, CV 0.43–0.62) and pulse group (*n* = 46, CV > 0.62) had similar grip strength. N.s., not significant. Data are presented as means ± SDs.

**Figure 3:**
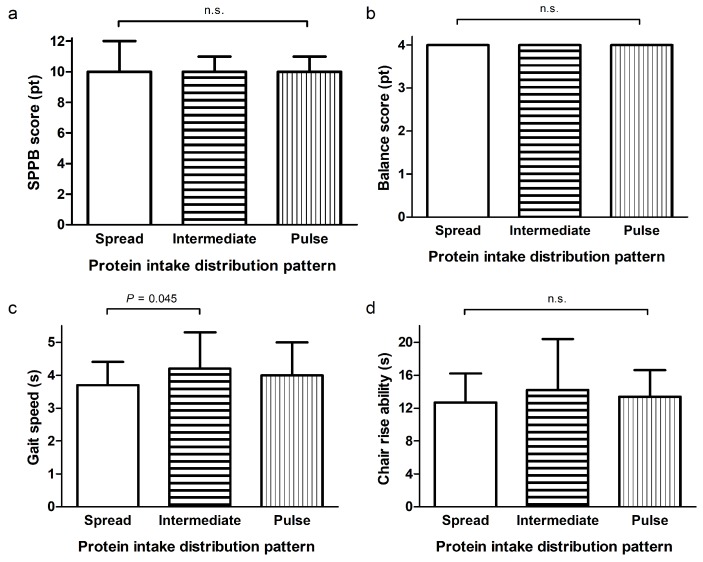
SPPB (Short Physical Performance Battery) total score (**a**); balance score (**b**); gait speed (**c**) and chair rise ability (**d**) of 3 groups based on distribution pattern of protein intake during the main meals determined with CV (coefficient of variance). Participants in the spread group (*n* = 46, CV < 0.43), intermediate group (*n* = 48, CV 0.43–0.62) and pulse group (*n* = 46, CV > 0.62) had similar scores for SPPB (**a**) and balance (**b**) and similar chair rise ability (**d**). Gait speed was significantly higher in the spread distribution group (3.7 ± 0.7) compared to the intermediate group (4.2 ± 1.1) *p* = 0.045. N.s., not significant. Data are presented as means ± SDs.

**Figure 4:**
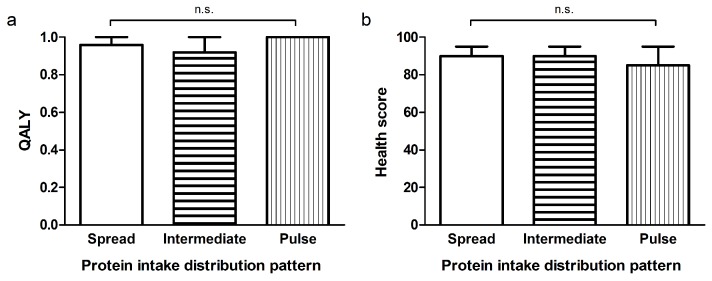
QALY (Quality-Adjusted Life Year) (**a**) and health score (**b**) of 3 groups based on distribution pattern of protein intake during the main meals determined with CV (coefficient of variance). Participants in the spread group (*n* = 46, CV < 0.43), intermediate group (*n* = 48, CV 0.43–0.62) and pulse group (*n* = 46, CV > 0.62) had similar QALY and health scores. N.s., not significant. Data are presented as means ± SDs.

Figure 1 presents the distribution of protein intake of each main meal across a day for the distribution-tertiles. Average protein intake of the spread group (CV < 0.43) varied less than 6.8 g between breakfast, lunch and dinner, whereas this range was 20.9 g and 29.3 g for the intermediate (CV 0.43–0.62) and pulse-feeding (CV > 0.62) group, respectively. The groups did not differ in age, sex, smoking behavior, level of education, body composition, dietary energy intake, carbohydrate intake, fat intake, vitamin D supplementation, grip strength (Figure 2), SPPB total score (Figure 3a), balance score (Figure 3b), chair rise ability time (Figure 3d), total physical activity, leisure time activity, household activity and quality of life (Figure 4a,b) (Table S1). Sports activity was significantly higher in the spread- and intermediate groups compared to the pulse group (∆0.7 METhr/day, *p* = 0.022 and ∆0.6 METhr/day, *p* = 0.044 respectively, Table S1) and gait speed was significantly higher in the spread distribution group compared to the intermediate group (∆0.5 s, *p* = 0.040, Figure 3c and Table S1). This was confirmed in the adjusted regression model in which a more spread protein distribution was related to a higher gait speed as opposed to the intermediate distribution group (*β* = −0.42, *p* = 0.035, Table 2).

### 3.4. Effect of Concurrent Physical Activity and Total Protein Intake

The interaction between physical activity and total protein intake was positively associated with QALY (*β* = 0.71, *p* = 0.049), whereas physical activity or total protein intake individually were not significantly related to QALY. No significant relation was found for the interaction between physical activity and total protein intake with grip strength, SPPB total score, balance score, chair rise ability time, gait speed (Table 2).

## 4. Discussion

In contrast to our hypotheses the results of our study show that in a sample of community-dwelling elderly people with a wide range of physical activity, total protein intake was not associated with muscle strength, physical function or quality of life. Nevertheless, a spread distribution of protein intake during the main meals as opposed to an intermediate feeding pattern was related to a higher gait speed. The interaction between physical activity and total protein intake was related to higher quality of life.

### 4.1. Total Protein Intake

In our study, we observed no association of total protein intake with SPPB scores, handgrip strength and quality of life. The absence of a positive effect of a higher total protein intake on these parameters might be explained by the fact that the contrast in total protein intake between the two groups was rather small and the average daily dietary intake of 1.1 ± 0.3 g protein/kg/day was well above the Recommended Dietary Allowance (RDA) of 0.8 g/kg/day. In our group analysis comparing lower versus higher total protein intake, we used the cut-off value of 1.0 g/kg/day, a value that has been suggested to be the RDA for total protein intake in the elderly [6,35,36]. Studies with larger sample sizes have indeed shown that intakes above 1.0 g/kg/day attenuate the decline in muscle mass and function in the elderly [37,38]. Moreover, Granic et al. [39] showed that an intake below 1.0 g/kg/day can negatively affect grip strength or physical functioning. On the contrary, a recent meta-analysis found no effect of protein or amino acid supplementation on muscle mass or strength in mostly non-frail elderly people with an average habitual total protein intake of 1.0 g/kg/day [40]. In our non-frail elderly population with on average a total protein intake well above the current RDA for protein, no additional beneficial effects for muscle strength or physical functioning were observed in those with a higher total protein intake.

### 4.2. Protein Intake Distribution

We determined tertiles of distribution based on a continuous measure (CV) by which we avoided the use of arbitrary cutoff values. In our adjusted regression model a spread protein intake pattern over the main meals was positively associated with gait speed as opposed to an intermediate pattern of intake. Gait speed in an elderly population is a strong predictor of survival [41] and is therefore an important marker of overall health. The proposed beneficial effects of a spread protein intake pattern over the main meals are in line with several studies in frail elderly people that have demonstrated benefits of a more evenly distributed protein intake on frailty, muscle protein synthesis and lean body mass [14,15,16,17]. Recommendations for a spread protein intake state that mealtime intake should be at least 25–30 g [42,43,44,45,46,47]. Our group with a spread protein intake had an average intake of 19 g, 21 g and 26 g for breakfast, lunch and dinner, respectively (Figure 1 and Table S1). The benefit of a spread distributed protein intake may even be higher with mealtime intakes reaching the 25–30 g threshold.

We found no positive effects of a pulse-feeding pattern compared to the intermediate cluster, whereas previous studies reported benefits of pulse feeding [12,13]. The fact that our CV for pulse-feeding pattern was lower than the CV defined by Cardon-Thomas et al. [48] and that the protein intake in our study was lower and less concentrated in one meal than presented in previous studies [12,13], suggests that our pulse feeding group did not completely comply to pulse feeding strategies used in other studies and therefore we should be cautious with the findings in our study that pulse feeding had no effect on any of the outcomes.

### 4.3. Concurrent Effect of Physical Activity and Total Protein Intake

The combination of physical activity and total protein intake was positively associated with overall quality of life in our adjusted model. Most studies that assessed the association between nutrition and quality of life, focus on nutritional status or malnutrition in frail or hospitalized populations [49,50,51]. While these studies do find a positive association between total protein intake and quality of life, no relationship was present in our study with total protein intake only. However, the combination of higher physical activity with higher total protein intake was positively associated with improved quality of life in physically active elderly people. A randomized controlled trial also found an increase in quality of life after participants performed resistance exercise training while increasing their protein intake [52]. These results emphasize the need of combining sufficient total protein intake with an active lifestyle in the elderly.

### 4.4. Strengths and Limitations

The strengths of this study were that we have a sample of community-dwelling elderly people with a high mean age (80+) and a broad range of physical activity levels (0–35 METhr/wk). Furthermore, we use a relevant set of validated outcomes, including objective measures (physical function, strength) and self-reported quality of life. Assessing physical activity with a questionnaire elicits less reliable measurements when compared to using activity monitors but these validated questionnaires provide a representative estimate of differences in physical activity between participants [31,32]. The cross-sectional design of the study limits the ability to assess causality. Furthermore, we have used data from two different studies that used different methods to assess dietary intake and physical activity. However, the dietary intake measures are comparable and we coded and calculated the dietary intake and physical activity data in the same way, which allowed us to combine the two studies and present a unique population of elderly people with a broad range of physical activity.

## 5. Conclusions

A higher total protein intake was not associated with improved physical outcome measures. A more spread protein intake during the main meals was related to a higher gait speed, an important measure of survival in the elderly. In addition, combining higher physical activity with higher total protein intake is related to a better quality of life, emphasizing the need for a higher total protein intake together with an active lifestyle in the elderly.

## Figures and Tables

**Table 1:** Differences between groups based on cutoff value of 1.0 g/kg/day.

Variables	Total Population	LPI <1.0 g/kg/day	HPI ≥1.0 g/kg/day	*p*-Value
	*n* = 140	*n* = 60	*n* = 80	
Age, year	83 (77–84)	83 (77–84)	83 (77–84)	0.98 ^2^
Male, n (%)	90 (64)	38 (63)	52 (65)	0.84 ^1^
Current smokers, n (%)	2 (1.4)	1 (2)	1 (1)	1.00 ^3^
Level of education				
Low, n (%)	14 (10.5)	6 (10)	8 (11)	
Intermediate, n (%)	78 (58.6)	36 (61)	42 (57)	0.88 ^1^
High/academic, n (%)	41 (30.8)	17 (29)	24 (32)	
**Body composition**				
Weight, kg				
Male	76.9 ± 9.4	79.5 ± 9.2	75.0 ± 9.3	0.025
Female	69.0 ± 12.6	74.8 ± 14.0	64.5 ± 9.4	0.003
BMI, kg/m^2^				
Male	25.9 ± 2.7	26.4 ± 2.8	25.5 ± 2.6	0.12
Female	26.4 ± 4.7	29.0 ± 5.1	24.4 ± 3.3	<0.001
**Dietary intake**				
Energy, kcal				
Male	2040 ± 370	1842 ± 336	2185 ± 325	<0.001
Female	1754 ± 396	1558 ± 344	1908 ± 369	0.001
Carbohydrate intake, en%	43.0 ± 6.0	43.0 ± 5.8	42.9 ± 6.2	0.91
Fat intake, en%	34.5 ± 5.6	34.8 ± 6.0	34.2 ± 5.4	0.61
Total protein intake, en%	16.4 ± 3.0	15.1 ± 2.3	17.4 ± 3.1	<0.001
Total protein intake, g	78.9 ± 18.9	64.7 ± 12.6	89.5 ± 15.6	<0.001
Total protein intake, g/kg/day	1.08 ± 0.29	0.84 ± 0.13	1.27 ± 0.23	<0.001
Animal-based protein, %	61.8 ± 9.2	59.5 ± 8.6	63.6 ± 9.3	0.009
Vitamin D supplementation, n (%)	30 (21)	13 (22)	17 (21)	0.73 ^1^
**Goldberg-score**				
EI/BMR	1.34 ± 0.28	1.17 ± 0.21	1.47 ± 0.25	<0.001
Underreporting, n (%)	5 (4)	5 (8)	0 (0)	
Within confidence limits, n (%)	135 (96)	55 (92)	80 (100)	0.013 ^3^
Overreporting, n (%)	0 (0)	0 (0)	0 (0)	
**Physical activity**				
Total activity, METhr/day	8.4 (5.1–13.7)	8.4 (5.6–12.6)	8.5 (5.0–14.5)	0.93 ^2^
Sports, METhr/day	0.4 (0.0–1.6)	0.4 (0.0–1.6)	0.4 (0.0–1.6)	0.93 ^2^
Household activities, METhr/day	2.5 (0.7–5.0)	2.4 (0.4–6.2)	2.6 (1.3–4.5)	0.69 ^2^
Leisure time, METhr/day	3.9 (1.8–7.3)	4.2 (1.7–7.3)	3.5 (1.8–7.4)	0.85 ^2^
**Muscle parameters**				
Grip strength, kg	32 ± 10	33 ± 10	32 ± 10	0.42
SPPB total score, pt	10 (9–11)	10 (9–11)	10.5 (9–11.3)	0.15 ^2^
SPPB balance score, pt	4 (3–4)	4 (3–4)	4 (3–4)	0.60 ^2^
SPPB gait speed, s	4.0 ± 1.0	4.0 ± 1.0	4.0 ± 1.0	0.76
SPPB chair rise ability time, s	13.4 ± 4.5	13.5 ± 3.8	13.4 ± 5.0	0.85
**Quality of Life**				
QALY	0.96 (0.86–1.00)	0.92 (0.86–1.00)	1.00 (0.86–1.00)	0.77 ^2^
Health score	90 (80–95)	85 (80–94)	90 (80–95)	0.21 ^2^

BMI, body mass index; EI/BMR, ratio of energy intake and basal metabolic rate; en%, energy percentage; g/kg/day, gram per kilogram of body weight per day; HPI, Higher total protein intake group; LPI, Lower total protein intake group; MET, metabolic equivalent of task; N, Newton; SPPB, Short Physical Performance Battery; QALY, Quality-Adjusted Life Year. Categorical values are given as number (percentage) of participants. Parametric continuous values are means ± SDs and non-parametric values are median (IQR). *p* values for differences between the two groups of total protein intake were derived by independent samples *t*-test unless otherwise indicated. ^1^ Derived by Chi-square test, ^2^ Derived by Kruskal-Wallis test. ^3^ Derived by Fisher’s exact test.

**Table 2:** Adjusted linear regression model * for SPPB total score, SPPB gait speed (s), SPPB chair rise (s), handgrip strength (kg) and QALY.

Variables	SPPB Total Score	Gait Speed	Chair Rise	Handgrip Strength	QALY ^‡^
Total protein intake (g/kg/day)	0.28	0.23	−1.78	0.76	−7.15
	(−0.89–1.45)	(−0.41–0.87)	(−5.10–1.43)	(−4.48–6.00)	(−17.59–3.29)
Protein distribution					
Spread (CV < 0.43)	0.62	**−0.42**	−1.46	2.36	−0.11
	(−0.09–1.33)	**(−0.80–−0.03)**	(−3.41–0.49)	(−0.80–5.52)	(−4.15–3.94)
Intermediate (CV 0.43–0.62) (*REF*)	1.00	1.00	1.00	1.00	1.00
Pulse (CV > 0.62)	0.17	−0.18	−0.99	1.73	0.07
	(−0.54–0.87)	(−0.56–0.21)	(−2.93–0.94)	(−1.40–4.86)	(−3.92–4.06)
Physical activity (METhr/day)	**0.06**	**−0.02**	−0.07	−0.02	0.48
	**(0.02–0.10)**	**(−0.05–−0.00)**	(−0.19–0.05)	(−0.21–0.18)	(−1.27–0.30)
Physical activity * total protein intake	n.s. §	n.s. §	n.s. §	n.s. §	**0.71**
					**(0.00–1.41)**
Animal-based protein (%)	0.02	−0.01	0.03	0.07	0.00
	(−0.01–0.06)	(−0.03–0.01)	(−0.07–0.12)	(−0.09–0.22)	(−0.20–0.19)

BMI, body mass index; CV, coefficient of variation; MET, metabolic equivalent of task; N.s., not significant; SPPB, Short Physical Performance Battery; QALY, Quality-Adjusted Life Year. * Adjusted for age, sex and BMI total protein intake, protein distribution, physical activity, interaction term between physical activity and total protein intake, animal-based protein. § Interaction term between physical activity and total protein intake was not significant and therefore not included in the final adjusted model. ^‡^ QALY was multiplied by 100. Bold values indicate *β* with *p*-value < 0.05.

## Supplementary Materials

The following are available online at http://www.mdpi.com/2072-6643/10/4/506/s1, Table S1. Tertiles based on distribution pattern of protein intake (CV).
