# Supplementary material for: Protein Intake and Distribution in Relation to Physical Functioning and Quality of Life in Community-Dwelling Elderly People: Acknowledging the Role of Physical Activity
Source: Nutrients. 2018 Apr 19;10(4):506. doi: 10.3390/nu10040506 (PMC5946291; doi:10.3390/nu10040506)
Supplement: Supplementary file 1 [file nutrients-10-00506-s001.pdf]

Table S1. Tertiles based on distribution pattern of protein intake (CV).

Variables	Spread (<0.43)	Intermediate (0.43-0.62)	Pulse (>0.62)	P-value
	n=46	*n*=48	*n*=46	
Age, y	83 (81-86)	82(75-83)	83(78-84)	0.10 [1]
Male, %	34 (74)	27 (56)	29 (63)	0.20 [1]
Current smokers, n (%)	1 (2)	1 (2)	0 (0)	0.68 [3]
Level of education				0.86 [1]
Low, n (%)	4 (9)	6 (13)	4 (9)	
Intermediate, n (%)	27 (61)	24 (52)	27 (63)	
High / academic, n (%)	13 (30)	16 (35)	12 (28)	
**Body composition**				
Weight, kg				
Male	76.3 ± 9.0	76.2 ± 11.3	78.3 ± 8.2	0.64
Female	65.9 ± 7.6	67.9 ± 12.7	72.9 ± 14.9	0.28
BMI, kg/m^2				
Male	25.6 ± 2.6	25.9 ± 3.0	26.3 ± 2.6	0.61
Female	25.0 ± 2.9	26.3 ± 5.0	27.6 ± 5.3	0.34
**Dietary intake**				
Energy, kcal				
Male	2032 ± 388	2115 ± 374	1981 ± 342	0.38
Female	1818 ± 411	1783 ± 405	1673 ± 383	0.58
Carbohydrate intake, en%	42.5 ± 6.2	43.6 ± 5.9	42.7 ± 5.9	0.34
Fat intake, en%	35.4 ± 6.5	34.3 ± 4.7	33.7 ± 5.5	0.66
Protein intake, en%	15.6 ± 2.4	17.0 ± 3.7	16.7 ± 2.6	0.06
Protein intake, g	76.8 ± 18.4	82.2 ± 19.7	77.5 ± 18.5	0.28
Protein intake at breakfast, g	18.9 ± 6.5	14.4 ± 5.4	9.8 ± 5.1	<0.001
Protein intake at lunch, g	21.4 ± 6.2	25.0 ± 9.7	19.6 ± 11.9	0.023
Protein intake at dinner, g	26.1 ± 8.5	35.3 ± 12.8	39.2 ± 15.7	<0.001
Protein intake, g/kg/day	1.05 ± 0.27	1.16 ± 0.31	1.04 ± 0.28	0.09
Animal-based protein, %	58.6 ± 8.5 *	62.5 ± 9.4	64.4 ± 9.0 *	0.009
Vitamin D supplementation, n (%)	9 (20)	11 (23)	10 (22)	0.89 [1]
**Goldberg-score**				
EI/BMR	1.35 ± 0.26	1.40 ± 0.28	1.28 ± 0.28	0.11
Underreporting, n (%)	0 (0)	2 (4)	3 (7)	
Within confidence limits, n (%)	46 (100)	46 (96)	43 (93)	0.29 [3]
Overreporting, n (%)	0 (0)	0 (0)	0 (0)	
**Physical activity**				
Total activity, METhr/day	8.2 (5.8-12.6)	9.0 (5.0-15.4)	8.4 (4.7-12.2)	0.89 [2]

Sports, METhr/day	0.7 (0.0-1.7)	0.6 (0.0-1.9)	0.0 (0.0-0.7)	0.045 [2]
Household activities, METhr/day	1.8 (0.3-3.6)	3.2 (1.3-6.1)	3.5 (0.6-5.7)	0.13 [2]
Leisure time, METhr/day	4.8 (2.5-8.2)	3.4 (1.3-7.1)	3.0 (2.0-6.7)	0.28 [2]
**Muscle parameters**				
Grip strength, N	34 ± 8	30 ± 11	33 ± 10	0.17
SPPB total score	11 (10-12)	10 (9-11)	10 (9-11)	0.22 [1]
SPPB balance score	4 (3-4)	4 (3-4)	4 (3-4)	0.90 [1]
SPPB gait speed, s	3.7 ± 0.7 *	4.2 ± 1.1 *	4.0 ± 1.0	0.045
SPPB chair rise ability time, s	12.7 ± 3.5	14.2 ± 6.2	13.4 ± 3.2	0.27
**Quality of Life**				
QALY	0.92 (0.88-1.00)	1.0 (0.86-1.00)	0.92 (0.86-1.00)	0.86
Health score	90 (80-95)	90 (80-95)	85 (75-95)	0.25

BMI, body mass index; CV, coefficient of variation; EI/BMR, ratio of energy intake and basal metabolic rate; en%, energy percentage; g/kg/d, gram per kilogram of body weight per day; MET, metabolic equivalent of task; N, Newton; SPPB, Short Physical Performance Battery; QALY, Quality-adjusted life year. Categorical values are given as number (percentage) of participants. Parametric continuous values are means ± SDs and non-parametric values are median(IQR). P values for differences between the two groups of protein intake were derived by independent samples t-test unless otherwise indicated. [1] Derived by Chi-square test, [2] Derived by Kruskal-Wallis test. [3] Derived by Fisher's exact test.* Significant difference between tertiles.
